# The GH5 1,4-β-mannanase from *Bifidobacterium animalis* subsp. *lacti*s Bl-04 possesses a low-affinity mannan-binding module and highlights the diversity of mannanolytic enzymes

**DOI:** 10.1186/s12858-015-0055-4

**Published:** 2015-11-11

**Authors:** Johan Morrill, Evelina Kulcinskaja, Anna Maria Sulewska, Sampo Lahtinen, Henrik Stålbrand, Birte Svensson, Maher Abou Hachem

**Affiliations:** Department of Biochemistry and Structural Biology, Center for Chemistry and Chemical Engineering, Lund University, P.O. Box 124, S-221 00 Lund, Sweden; Enzyme and Protein Chemistry (EPC), Department of Systems Biology, Technical University of Denmark (DTU), Søltofts Plads, building 224, DK-2800 Kgs Lyngby, Denmark; Active Nutrition, DuPont Nutrition & Health, Sokeritehtaantie 20, 02460 Kantvik, Finland; Current address: Biochemistry and Bioprocessing, Department of Food Science, University of Copenhagen, Rolighedsvej 30, DK-1958 Fredriksberg C, Denmark

**Keywords:** *Bifidobacterium*, Carbohydrate-binding module, Gut microbiota, Mannan, Probiotic bacteria, Surface plasmon resonance

## Abstract

**Background:**

β-Mannans are abundant and diverse plant structural and storage polysaccharides. Certain human gut microbiota members including health-promoting *Bifidobacterium* spp. catabolize dietary mannans. Little insight is available on the enzymology of mannan deconstruction in the gut ecological niche. Here, we report the biochemical properties of the first family 5 subfamily 8 glycoside hydrolase (GH5_8) mannanase from the probiotic bacterium *Bifidobacterium animalis* subsp. *lactis* Bl-04 (*Bl*Man5_8).

**Results:**

*Bl*Man5_8 possesses a novel low affinity carbohydrate binding module (CBM) specific for soluble mannan and displays the highest catalytic efficiency reported to date for a GH5 mannanase owing to a very high *k*_cat_ (1828 ± 87 s^-1^) and a low *K*_m_ (1.58 ± 0.23 g · L^-1^) using locust bean galactomannan as substrate. The novel CBM of *Bl*Man5_8 mediates increased binding to soluble mannan based on affinity electrophoresis. Surface plasmon resonance analysis confirmed the binding of the CBM10 to manno-oligosaccharides, albeit with slightly lower affinity than the catalytic module of the enzyme. This is the first example of a low-affinity mannan-specific CBM, which forms a subfamily of CBM10 together with close homologs present only in mannanases. Members of this new subfamily lack an aromatic residue mediating binding to insoluble cellulose in canonical CBM10 members consistent with the observed low mannan affinity.

**Conclusion:**

*Bl*Man5_8 is evolved for efficient deconstruction of soluble mannans, which is reflected by an exceptionally low *K*_m_ and the presence of an atypical low affinity CBM, which increases binding to specifically to soluble mannan while causing minimal decrease in catalytic efficiency as opposed to enzymes with canonical mannan binding modules. These features highlight fine tuning of catalytic and binding properties to support specialization towards a preferred substrate, which is likely to confer an advantage in the adaptation to competitive ecological niches.

**Electronic supplementary material:**

The online version of this article (doi:10.1186/s12858-015-0055-4) contains supplementary material, which is available to authorized users.

## Background

β-Mannans (hereafter mannans) are abundant polysaccharides playing diverse roles in plants including energy storage in seed endosperms, *e.g*. carob and guar seeds, legumes, coconuts and coffee beans [1], or structural support in the hemicellulose cell wall matrix [2], where they can constitute up to 25 % of the dry mass in softwood. The biochemical details of enzymatic mannan depolymerization have received increasing attention in recent years due to wide interest within the biofuel and biorefinery areas [3, 4]. Mannans, however, are also present in human nutrition, either as cell wall components in cereal grains and some fruits and vegetables such as kiwi, apple and tomato [5–8] or as common hydrocolloid food additives used as thickeners or to adjust texture [9, 10]. Mannans are not known to be hydrolyzed by human digestive enzymes and thus offer a potential resource to mannanolytic gut bacteria. The fermentation of guar gum (GG) galactomannan has been demonstrated in the human gut [11] and intake of partial hydrolysates of this polysaccharide stimulated the proliferation of *Bifidobacterium* spp. in humans [12] and mice [13]. However, insight into the microbial strategies and enzymes mediating mannan degradation in the human gut lags behind.

Mannans occur as insoluble crystalline polymers of β-1,4-linked mannosyl residues in seeds, such as ivory nut mannan (INM) or as soluble heteropolymeric glucomannans consisting of alternating β-1,4-linked glucosyl and mannosyl backbone *e.g*. in corms of *Amorphophallus konjac*. Mannan can be substituted to different degrees with α-1,6-linked galactosyl sidechains as in carob (locust bean) and guar gums and in glucomannan from softwood, which also has acetyl decorations [14]. Konjac glucomannan (KGM), locust bean (LBG) and GG galactomannans are the main mannans used as food additives [9, 10].

The concerted action of several backbone- and sidechain-degrading enzymes is required for depolymerization of mannans. The precise number of enzymatic activities varies with the substrate structure, but endo-β-1,4-mannanases (EC 3.2.1.78) that hydrolyze internal backbone β-1,4-linkages are central in mannan degradation. β-Mannanases are assigned in glycoside hydrolase (GH) families 5, 26 and 113 in the Carbohydrate-Active enZYmes (CAZy) database [15]. Many bacterial mannanases cluster in subfamily 8 of GH5 (GH5_8) according to a phylogeny-based assignment [16]. Mannanases of GH5 employ a double displacement mechanism with the retention of anomeric configuration [17]. Some mannanases contain carbohydrate-binding modules (CBMs) that have been ascribed diverse roles including targeting enzymes to polysaccharides, increasing the local substrate concentration, or conferring processivity [18, 19].

The present study focuses on the widely utilized and clinically well-documented probiotic bacterium *Bifidobacterium animalis* subsp. *lactis* Bl-04 [20]. Genome analysis identified a gene encoding a mannanase comprising a GH5 catalytic module joined to a CBM10. Members of CBM10 were previously found to bind to insoluble microcrystalline cellulose [21] and insoluble mannan [22]. Furthermore, we show that the GH5 mannanase (*Bl*Man5_8) from *Bifidobacterium animalis* subsp. *lactis* Bl-04, which is conserved within *Bifidobacterium animalis* subsp. *lactis*, displays the highest catalytic efficiency reported to date for a GH5 β-mannanase owing to a combination of very high *k*_cat_ and low *K*_m_. The CBM10 of this enzyme is the first described low-affinity mannan binding module and it forms a novel CBM10 subfamily together with close homologues. The distinct differences in the biochemical properties of this enzyme as compared to characterized β-mannanases from gut microbiota illustrate the diversity of mannan utilization strategies, which is likely to be important in adaptation to the highly competitive gut niche.

## Methods

### Carbohydrates

Cellotetraose, locust bean gum (LBG), microcrystalline cellulose (Avicel) and hydroxyethyl cellulose (HEC) are from Sigma-Aldrich (St. Louis, MO, USA); mannotriose (M_3_), mannotetraose (M_4_), mannopentaose (M_5_), mannohexaose (M_6_), low-viscosity locust bean gum (LBG-lv), ivory nut mannan (INM) and konjac glucomannan (KGM) are from Megazyme (Bray, Ireland); guar gum (GG) is from Carl Roth (Karlsruhe, Germany). The compositions of the polysaccharides are listed in Additional file 1.

### Cloning

*Bifidobacterium animalis* subsp. *lactis* Bl-04 chromosomal DNA [23] was used to clone the locus Balac_1450 (GenBank accession number ACS46797) encoding a β-mannanase of glycoside hydrolase family 5 subfamily 8 (GH5_8). The PCR-amplified gene fragment encoding the full-length mature β-mannanase lacking the N-terminal signal peptide (amino acid residues 1–29 as predicted by Signal P v.4.0, http://www.cbs.dtu.dk/services/SignalP/), referred to as *Bl*Man5_8, was cloned within the *Nhe*I and *Bam*HI restriction sites in pET28a(+) (Novagen, Darmstadt, Germany) using a primer pair (Additional file 2) to yield the plasmid pET28a-*Bl*Man5_8. This plasmid, encoding the enzyme fused to an N-terminal hexa-histidine purification tag, was transformed first into *Escherichia coli* TOP10 (Life Technologies, Grand Island, NY, USA). The presence of the insert was verified by sequencing and restriction analysis. The construct encoding the truncated enzyme *Bl*Man5_8-ΔCBM10 lacking the C-terminal CBM10, was generated by site-directed mutation of Ser^338^, which is located approximately in the middle of the linker sequence (G^330^GGSNSGGSGNTGGNSGTTDDG^351^) between the catalytic and the binding modules, into a stop codon (QuikChange mutagenesis kit; Agilent Technologies, Santa Clara, CA, USA) and the primer pair in Additional file 2 to yield pET28a-*Bl*Man5_8-ΔCBM10. The mutation was verified by full sequencing. The plasmids encoding *Bl*Man5_8 and *Bl*Man5_8-ΔCBM10 were transformed into *E. coli* BL21 (DE3) for production.

### Enzyme production and purification

Production of *Bl*Man5_8 and *Bl*Man5_8-ΔCBM10 was carried out in 2 L baffled shake flasks. Overnight cultures were used to inoculate 1 L LB medium containing 10 mM glucose and 50 μg · mL^−1^ kanamycin and grown at 30 °C to an OD_*600*_ of 0.5. Protein expression was induced with 100 μM IPTG and growth was continued for 5 h. For *Bl*Man5_8, the cells were harvested by centrifugation, resuspended in binding buffer (10 mM HEPES, 0.5 M NaCl, 10 % glycerol, 15 mM imidazole, pH 7.4) with added protease inhibitor cocktail (Roche Diagnostics, Indianapolis, IN) and lysed using a high pressure homogenizer, centrifuged and filtered through a 0.45-μm filter. For *Bl*Man5_8-ΔCBM10, the cells were lysed using the BugBuster™ Protein Extraction Reagent (Novagen), according to the manufacturer’s recommendations. Clarified *Bl*Man5_8 and *Bl*Man5_8-ΔCBM10 lysates were applied onto a 5-mL HisTrap HP (GE Healthcare, Uppsala, Sweden), washed with binding buffer, and eluted with a gradient formed with 400 mM imidazole in 10 mM HEPES, 0.5 M NaCl, 10 % glycerol, pH 7.4. Eluted fractions were analyzed by SDS-PAGE and for β-mannanase activity as described below. Pure fractions were pooled and concentrated (10 kDa cut-off ultrafiltration units; Amicon), and the histidine tag was cleaved off using human plasma thrombin (Calbiochem, San Diego, CA, USA) according to the manufacturer’s instructions. Cleaved enzymes were recovered after passing through a binding buffer pre-equilibrated 1 mL HisTrap HP column (GE Healthcare). The concentrated enzyme samples were further purified to electrophoretic homogeneity by chromatography on a HiLoad Superdex G75 26/60 column (GE Healthcare) eluted with 10 mM HEPES, pH 7.0 in 1.2 column volumes. The pure enzyme samples were concentrated as above and stored at 4 °C until further use.

### Basic enzymatic properties and enzyme stability

β-Mannanase activity was measured towards 2.5 g · L^−1^ LBG at 37 °C for 10 min in 400 μL standard assay buffer (40 mM phosphate-citrate buffer, pH 6.0, with 0.005 % (v/v) Triton X-100) with appropriately diluted enzyme. The substrate preparation and reducing end 3,5-dinitrosalicylic acid (DNS) assay were performed as previously reported [24, 25]. The pH dependence of activity was initially determined using 6.4 nM *Bl*Man5_8 with LBG in 100 mM Britton-Robinson buffers at pH 2 − 10 and then refined in 40 mM phosphate-citrate at pH 5 − 7. To assess storage stability as function of pH, 1.28 μM *Bl*Man5_8 was incubated at 4 °C for four days in 100 mM Britton-Robinson buffers at pH 2 − 10, thereafter diluted to 3.2 nM in standard assay buffer, and residual β-mannanase activity was determined as described above. The dependence of enzyme activity on temperature was determined for 6.4 nM *Bl*Man5_8 with LBG in standard assay buffer, in the range 25 − 80 °C.

The conformational stability of 12.6 μM *Bl*Man5_8 in 10 mM MES buffer, pH 6.5 was assessed using differential scanning calorimetry (DSC) between 15 °C and 90 °C at 1 °C · min^−1^ in a MicroCal VP-DSC calorimeter. The enzyme was scanned twice with cooling and pre-equilibration in between to assess the reversibility of thermal transitions. The dialysis buffer was used as a reference and the reference and baseline corrected thermograms were analyzed using Origin7 with a DSC add-on provided with the instrument.

### Substrate specificity

The soluble substrates LBG, LBG-lv, GG and KGM were prepared as previously described [25]. The insoluble substrates INM and Avicel were washed three times in water, twice for 2 h and once overnight, with a 15:1 volume to mass ratio, followed by 3 h wash in buffer. The specific activity of *Bl*Man5_8 towards LBG-lv and KGM (2.5 g · L^−1^, 10 min incubation), as well as GG (2.5 g · L^−1^, 2.5 h incubation), INM (5 g · L^−1^, 45 min incubation) and Avicel (10 g · L^−1^, 3 h incubation) was measured using the standard activity assay.

### Enzyme kinetics

LBG-lv (0.45 − 9.0 g · L^−1^) was hydrolyzed by 1 nM *Bl*Man5_8 or *Bl*Man5_8-ΔCBM10 at 37 °C in standard assay buffer as measured by DNS (see above). The initial reaction rates were determined and the kinetic parameters *k*_cat_ and *K*_m_ were determined by fitting the Michaelis-Menten equation to the data using Prism 6 (GraphPad Software, La Jolla, CA, USA). Experiments were performed in duplicates.

For kinetic analysis of M_5_ hydrolysis, 0.25 nM *Bl*Man5_8 was incubated with 0.25–10 mM M_5_ at 37 °C in standard assay buffer. Samples were withdrawn at 0, 5, 10, 15 and 20 min, and boiled for 5 min. The hydrolysis products of M_5_ were analyzed by HPAEC-PAD using a DX-500 system (Thermo Scientific Dionex, Sunnyvale, CA, USA). A CarboPac™ 100 column and guard column were used with a mobile phase of 78 mM sodium hydroxide at a flow rate of 1.0 mL · min^-1^. The decrease in M_5_ concentration is stoichiometrically equivalent to the increase in concentrations of degradation products. These concentrations were calculated and used to determine *k*_cat_ and *K*_m_ as described [26].

### Mannan hydrolysis action patterns

The product profiles for 5.3 nM *Bl*Man5_8 or *Bl*Man5_8-ΔCBM10 towards 2.5 g · L^−1^ INM or LBG in standard assay buffer at 37 °C at 0 min, 30 s, 5, 15 and 30 min, as well as 2 and 24 h, were analyzed by HPAEC-PAD (ICS-5000 system; Thermo Scientific Dionex, Sunnyvale, CA, USA). A CarboPac™ PA-200 column and guard column were used with a mobile phase of 78 mM sodium hydroxide and a linear gradient of 0 − 50 mM sodium acetate at a flow rate of 0.5 mL · min^−1^.

### Binding to polysaccharides and oligosaccharides

Qualitative screening of binding of *Bl*Man5_8 and *Bl*Man5_8-ΔCBM10 to soluble polysaccharides was analysed using affinity electrophoresis as previously described [27]. The electrophoretic separation of 2 μg of either protein was performed at 4 °C and 45 V for 20 h in 50 mM Tris-Borate, pH 8.7 and in 12 % polyacrylamide gels without (control) or with 0.1, 0.5 or 2.5 g · L^−1^ KGM, as well as 2.5 g · L^−1^ hydroxyethyl cellulose (HEC). Quantitative binding analysis of *Bl*Man5_8 and *Bl*Man5_8-ΔCBM10 to LBG-lv was done using affinity electrophoresis [27, 28] at 100 V for 4 h in gels without (control) or with 0.05, 0.10 or 0.25 g · L^−1^ LBG-lv, as described above. The apparent dissociation constant (*K*_d_) for LBG-lv was calculated by nonlinear regression using Prism 6 (GraphPad Software, La Jolla, CA, USA). Binding to insoluble INM and Avicel was measured by adding 50 μl of appropriately diluted enzyme to 400 μl of 0 (negative control), 0.5, 5, 50 or 100 g · L^−1^ of either substrate suspended in standard assay buffer. The slurry was incubated at 4 °C for 1 h with gentle shaking, centrifuged at 16 000 × g for 10 min and the mannanase activity in the supernatant was assayed as described above.

Surface plasmon resonance (SPR) analysis was performed using a Biacore T100 (GE Healthcare) to assess binding to manno-oligosaccharides and cellotetraose. Enzymes diluted to 40 μg · mL^−1^ in 10 mM sodium acetate, pH 3.9, were immobilized onto a CM5 chip (GE Healthcare) using a standard amine coupling protocol to a density of 3999 and 2879 response units (RU) for *Bl*Man5_8 or *Bl*Man5_8-ΔCBM10, respectively. Binding analysis was performed in triplicates at 25 °C in 10 mM MES, 150 mM NaCl, pH 6.0, 0.005 % (v/v) P20 (GE Healthcare) running buffer at 30 μL · min^−1^ with association and dissociation times of 60 and 30 s, respectively, at seven concentrations of M_4_ (0.5 − 25 mM), M_5_ (0.05 − 25 mM), M_6_ (0.05 − 12.5 mM) and cellotetraose (0.05 − 25 mM). A one-binding-site model was fit to the blank and reference cell-corrected steady state response data to determine the dissociation constant *K*_d_ and the saturation binding level *R*_max_, which was used to estimate the molar stoichiometry (*n*) [29] of oligosaccharide binding to immobilized enzymes that retain binding activity.

### Action pattern analysis using mannopentaose

For analysis of the productive binding modes of M_5_, enzymatic reactions were carried out in ^18^O labeled water (H_2_^18^O 97 % pure from Sigma-Aldrich, St. Louis, MO, USA), as described [26, 30] at 8 °C with 1 mM M_5_ and 20 nM *Bl*Man5_8 in 1 mM sodium phosphate, pH 6.0 in 93 % H_2_^18^O. Samples were incubated for 0 − 24 h and analyzed with MALDI-TOF MS. Aliquots of 0.5 μL were withdrawn at each time point, spotted directly on a MALDI plate, covered with 0.5 μL matrix (10 g · L^−1^ 2,5-dihydroxybenzoic acid (DHB) in water) and dried under warm air. This analysis combined with HPAEC-PAD quantification of M_3_ and M_4_ products of M_5_ hydrolysis was used to determine the frequency of productive binding modes of M_5_ as previously described [26].

### Transglycosylation product formation from M_5_

For analysis of transglycosylation 5 mM M_5_ was incubated with 1.6 nM *Bl*Man5_8 in standard assay buffer for 0–24 h. Samples were analyzed with MALDI-TOF MS as described above.

### Sequence analyses and homology modeling

The sequence of *Bl*Man5_8 was analyzed to identify catalytic and ancillary modules using dbCAN [31], a CAZy-based database [15] for automated carbohydrate-active enzyme annotation. The sequence of the *Bl*Man5_8 catalytic module was aligned with 69 sequences listed as GH5_8 in CAZy [15], as well as 12 characterized β-mannanases from GH5 subfamilies 7, 8 and 10, using MAFFT 7 [32]. The CBM10 of *Bl*Man5_8 was aligned with 83 predicted CBM10 sequences retrieved using dbCAN, after removing two sequences that appeared fragmented and three sequences that displayed E-values above 0.02. These sequences were aligned using MAFFT 7 and a phylogenetic tree was calculated using the unweighted pair group method with arithmetic mean using standard settings accessed on the CBRC server (http://mafft.cbrc.jp/alignment/server/phylogeny.html) and rendered using Dendroscope 3 [33]. The crystal structure of the *Thermobifida fusca* mannanase *Tf*Man5A (PDB ID: 2MAN) [34] served as template to model the 39 % amino acid sequence identical catalytic module of *Bl*Man5_8, using MODELLER [35]. The homology model was evaluated with MolProbity [36], showing that 93.29 % of all residues were in Ramachandran favored regions. The model had a clash score of 72.89, defined as the number of unfavorable (≥0.4 Å) steric overlaps per 1000 atoms [37]. Evaluation with ProQ [38] gave a predicted LGscore of 5.903 and a predicted MaxSub of 0.266, indicating good model quality.

## Results

### Basic properties of *Bl*Man5_8

*Bl*Man5_8 is predicted to be an extracellular modular β-mannanase comprising a catalytic module of glycoside hydrolase family 5 subfamily 8 (GH5_8) joined to a C-terminal carbohydrate-binding module of family 10 (CBM10) by a typical linker sequence plausibly starting from Gly^330^ to Gly^351^ (see sequence in Methods). The position of the truncation (Ser^338^) was chosen as to avoid destabilisation of the catalytic module due to possible loss of interactions with the first few residues in the linker. The full-length *Bl*Man5A and the truncated *Bl*Man5_8-ΔCBM10 were produced and purified to electrophoretic homogeneity. Both enzyme forms migrated in accord with their theoretical molecular mass of 37.9 kDa and 33.8 kDa, respectively. The lack of activity towards microcrystalline cellulose (Avicel) and the high activity towards mannans (Table 1) confirmed the β-mannanase specificity of *Bl*Man5_8.

**Table 1:** Specific activity of *Bl*Man5_8 towards soluble and insoluble polysaccharides

Substrate	Specific activity (kat · mol^−1^)	Specific activity (U · mg^−1^)
LBG	1213	1920
LBG-lv	872	1380
GG	n.d.^a^	n.d.^a^
KGM	1592	2520
INM	76	120
Avicel	n.d.^a^	n.d.^a^

^a^ Not detected

*Bl*Man5_8 has highest activity at pH 6.0 and retains approximately 75 % of its maximal activity between pH 5 and 7 using LBG as a substrate (Additional file 3A). After incubation for 4 days at 4 °C, the enzyme retained 100 % of its initial activity at pH 8.0, and approximately 75 % at pH 6.0 (Additional file 3B). *Bl*Man5_8 displayed highest β-mannanase activity at 55 °C and pH 6.0 (Additional file 4A), corresponding to an Arrhenius activation energy of 69.5 kJ · mol^−1^ (Additional file 4B). The unfolding temperature of *Bl*Man5_8 was determined to 55.9 °C by differential scanning calorimetry (DSC) analysis asserting the structural integrity of the enzyme. The unfolding thermogram featured a single transition (Additional file 5), suggesting that the unfolding of the two modules of *Bl*Man5_8 is overlapping.

### Substrate specificity and kinetics

Activity of *Bl*Man5_8 was highest towards the soluble KGM followed by LBG and low-viscosity LBG (LBG-lv) (Table 1). Activity towards insoluble INM was only 6 % of that on LBG, and no activity towards GG could be measured (Table 1). The apparent kinetic parameters for *Bl*Man5_8 and *Bl*Man5_8-ΔCBM10 were determined for LBG-lv (Table 2; Additional file 6A) and marginal increases in *K*_m_ and *k*_cat_ were observed as a result of deleting the CBM10. Apparent kinetic parameters were also determined towards mannopentaose (M_5_) for *Bl*Man5_8 (Table 2; Additional file 6B). The enzyme displayed no activity towards mannotriose (M_3_), and very low activity towards mannotetraose (M_4_), but was highly active on larger manno-oligosaccharides as analyzed by high-performance anion exchange chromatography with pulsed amperometric detection (HPAEC-PAD) (data not shown).

**Table 2:** Kinetic parameters for *Bl*Man5_8 on LBG-lv and M_5_

Substrate	Enzyme	*k*_cat_	*K*_m_	*k*_cat_/*K*_m_
LBG-lv	*Bl*Man5_8	1828 ± 87 s^−1^	1.58 ± 0.23 g · L^−1^	1157 ± 177 L · s^−1^ · g^−1^
	*Bl*Man5_8-ΔCBM10	2005 ± 179 s^−1^	1.75 ± 0.47 g · L^−1^	1146 ± 324 L · s^−1^ · g^−1^
M_5_	*Bl*Man5_8	97 ± 2 s^−1^	1.09 ± 0.11 mM	89 ± 9 s^−1^ mM^−1^

### Product profiles, transglycosylation and productive binding frequency analysis

**Fig. 1:**
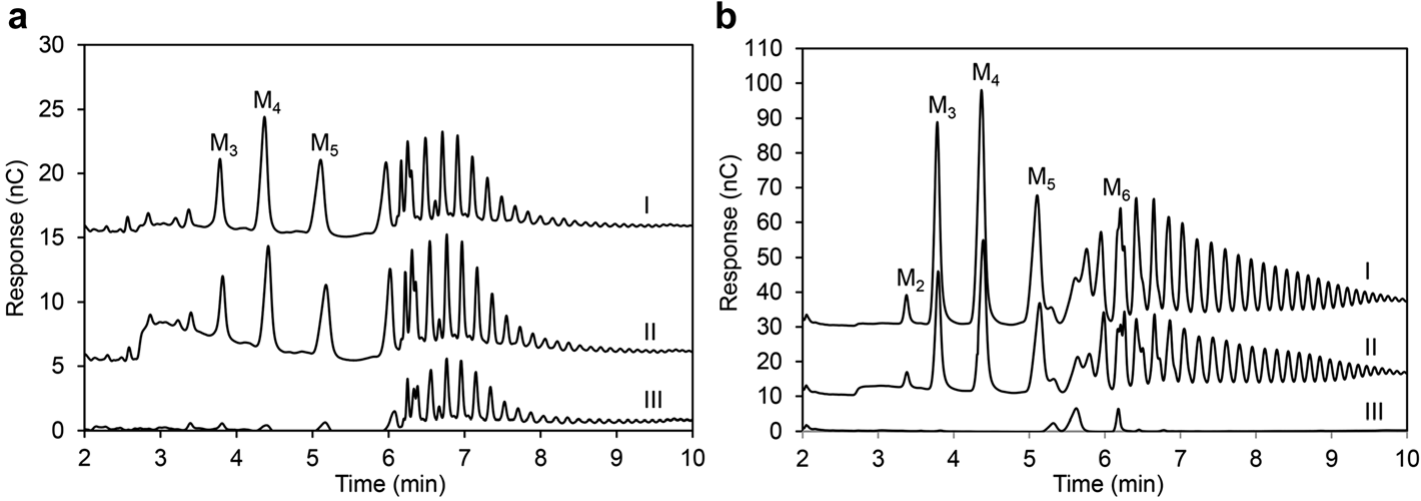
Initial products from INM and LBG. Manno-oligosaccharide products formed after 30 s of **a** INM and **b** LBG hydrolysis by *Bl*Man5_8 as measured using HPAEC-PAD analysis. The chromatograms from *Bl*Man5_8-ΔCBM10 (I), *Bl*Man5_8 (II) and the substrate before hydrolysis (III)

**Fig. 2:**
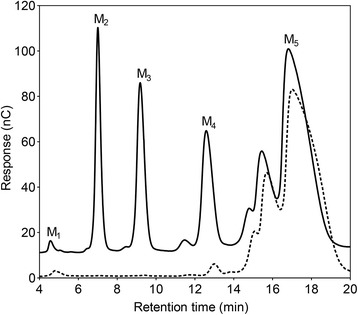
Product formation from M_5._ Products after 20 min of reaction of 2 mM M_5_ with *Bl*Man5_8 (solid line) compared to the substrate before hydrolysis (dashed line) as analyzed by HPAEC-PAD. The solid line is offset by 10 nC on the y axis for clarity

**Fig. 3:**
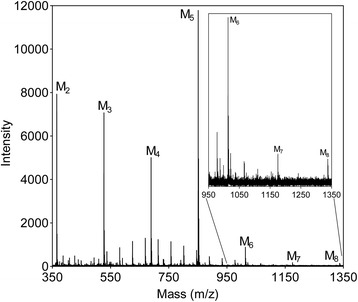
Transglycosylation product formation. Mass-spectrum (MALDI-TOF MS) of the product formation by *Bl*Man5_8 after 2 h of incubation with 5 mM M_5_. The inserted mass-spectrum is a close that shows the mass-spectrum from m/z 950 to1350

**Fig. 4:**
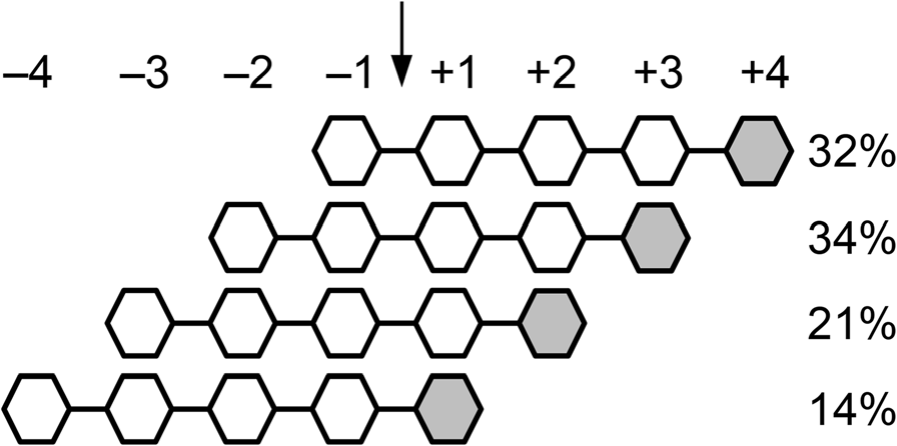
Relative productive binding frequencies for M_5_ to *Bl*Man5_8. Percentages are calculated by combining HPAEC-PAD product quantification with analysis of ^18^O labeling using MALDI-TOF MS. Numbers from −4 to +4 designate substrate-binding subsites. The arrow indicates the scissile bond hydrolyzed by the enzyme. The grey sugar unit indicates the reducing end of M_5_

The hydrolysis profiles of LBG and INM by *Bl*Man5_8 and *Bl*Man5_8-ΔCBM10 were analyzed using HPAEC-PAD. Both enzyme variants produced M_3_, M_4_ and M_5_ as the main initial hydrolysis products from both LBG and INM, along with some mannobiose (M_2_) (Fig. 1). The main products of *Bl*Man5_8 from M_5_ were M_2_, M_3_ and M_4_. The formation of 10-fold higher M_4_ compared to mannose (M_1_) (Fig. 2) is indicative of transglycosylation activity, which was supported by the detection of mannohexaose (M_6_), mannoheptaose (M_7_) and mannooctaose (M_8_) transglycosylation products by mass-spectrometry following incubation with M_5_ (Fig. 3). All four productive M_5_ binding modes were observed, albeit with higher frequency (66 %) for binding modes where the larger part of the substrate was bound at the aglycone binding subsites (Fig. 4).

### Binding to polysaccharides and oligosaccharides

Based on retardation in affinity electrophoresis, *Bl*Man5_8 bound to LBG-lv (Additional files 7–8) with an apparent *K*_d_ of 0.31 g · L^−1^, while the affinity of *Bl*Man5_8-ΔCBM10 was too low to be determined due to lack of change in relative retardation as a function of increasing the LBG concentration. Neither the full-length nor the truncated enzyme displayed binding to soluble hydroxyethyl cellulose (HEC), insoluble cellulose (Avicel) or insoluble mannan (INM) in this analysis.

SPR is a particularly powerful tool to quantify low affinity interactions as observed above for *Bl*Man5_8 binding to mannan. Ideally, CBM binding analysis would be carried out on an isolated CBM. In our case, however, the small size of the CBM10 and the presence of only a single lysine residue (used for random amine coupling or biotinylation), renders the immobilization of the protein to sufficient levels challenging. To circumvent this limitation and avoid using this single lysine residue for immobilization with risk of affecting the function of the module, the binding analysis was done using immobilized full-length enzyme and *Bl*Man5_8-ΔCBM10 to evaluate the contribution of the CBM10 to binding. Only the full-length enzyme showed affinity towards M_4_ based on the SPR analysis, which suggested that the CBM10 mediated binding to M_4_ (Table 3). The apparent binding stoichiometry of both M_5_ and M_6_ was approximately twice for the full-length enzyme as compared to the isolated catalytic module (Table 3), reflecting binding of these ligands to both the GH5 catalytic module and the CBM10. No binding to cellotetraose was measured with either enzyme variant.

**Table 3:** Surface plasmon resonance binding analysis of *Bl*Man5_8 to oligosaccharides

Enzyme	Substrate	*K*_d_ (mM)	*n* ^a^
*Bl*Man5_8	M_4_	5.1 ± 0.8	0.78
	M_5_	3.7 ± 0.3	0.94
	M_6_	0.58 ± 0.05	0.92
	Cellotetraose	n.d.^b^	–
*Bl*Man5_8-ΔCBM10	M_4_	n.d.^b^	–
	M_5_	1.5 ± 0.3	0.36
	M_6_	0.24 ± 0.42	0.53
	Cellotetraose	n.d.^b^	–

^a^ Estimated stoichiometry of binding to the immobilized protein

^b^ Not detected

### Sequence analyses and homology modeling

**Fig. 5:**
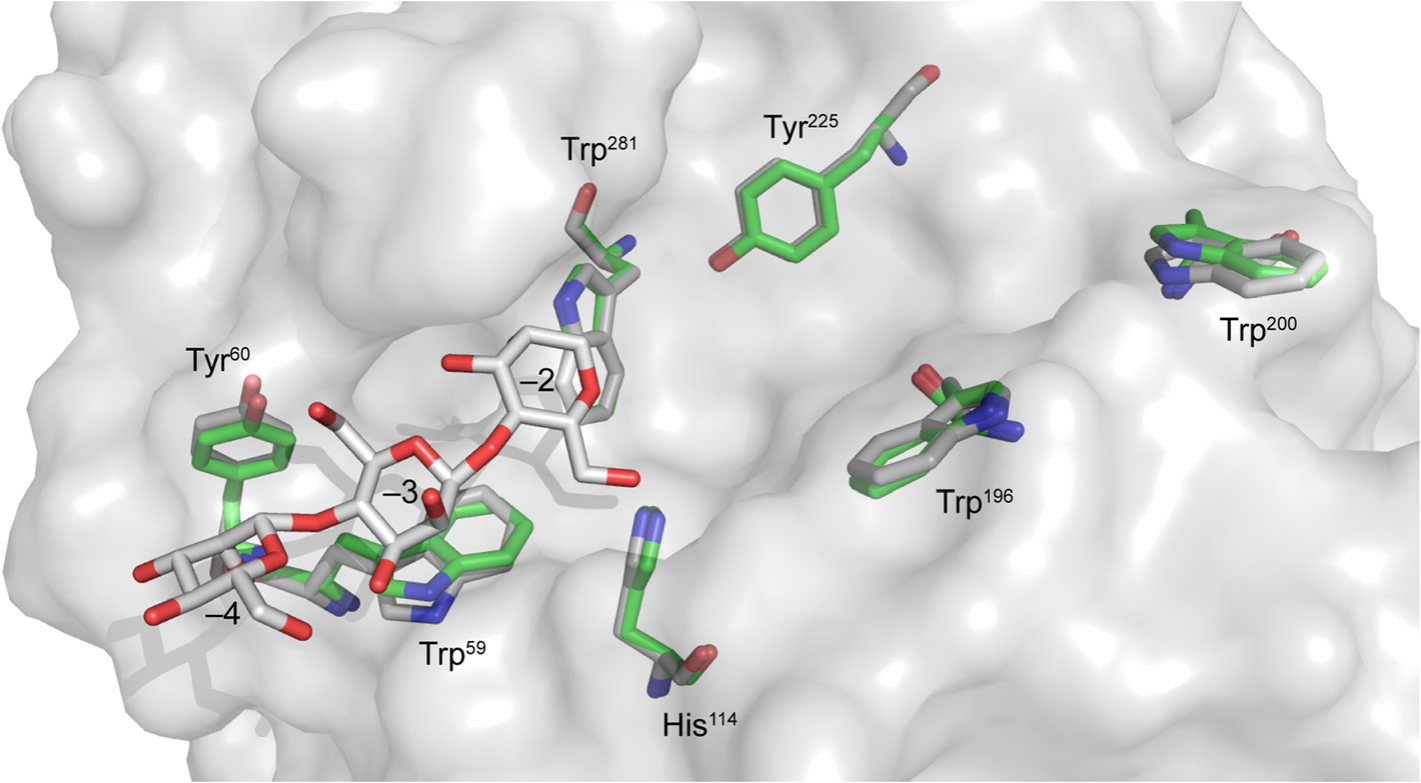
Modeling of putative subsite residues in *Bl*Man5_8. Conserved aromatic residues in the active site of *Bl*Man5_8 are visualized (green) by homology modeling using the structure from *Tf*Man5A (PDB ID: 1BQC) as template (grey). A semi-transparent molecular surface is shown to depict the topology of the active site of *Tf*Man5A. The conserved aromatic residues are designated with *Bl*Man5_8 numbering and the co-crystallized M_3_, accommodated at subsites –4 through –2 in *Tf*Man5A, is shown in white

*Bl*Man5_8 contains the seven amino acids that are conserved in family GH5 [39] (Additional file 9). The homology model of *Bl*Man5_8 revealed that residues interacting with the substrate in subsites –4 through –2 are conserved between *Bl*Man5_8 and *Tf*Man5A [34] (Fig. 5). There are also several aromatic residues in the putative aglycone binding region of *Bl*Man5_8 including Trp^196^ and Trp^225^ which are invariant in GH5_8 as well as Trp^200^ which is conserved in 77 % of GH5_8 sequences (Additional file 10).

**Fig. 6:**
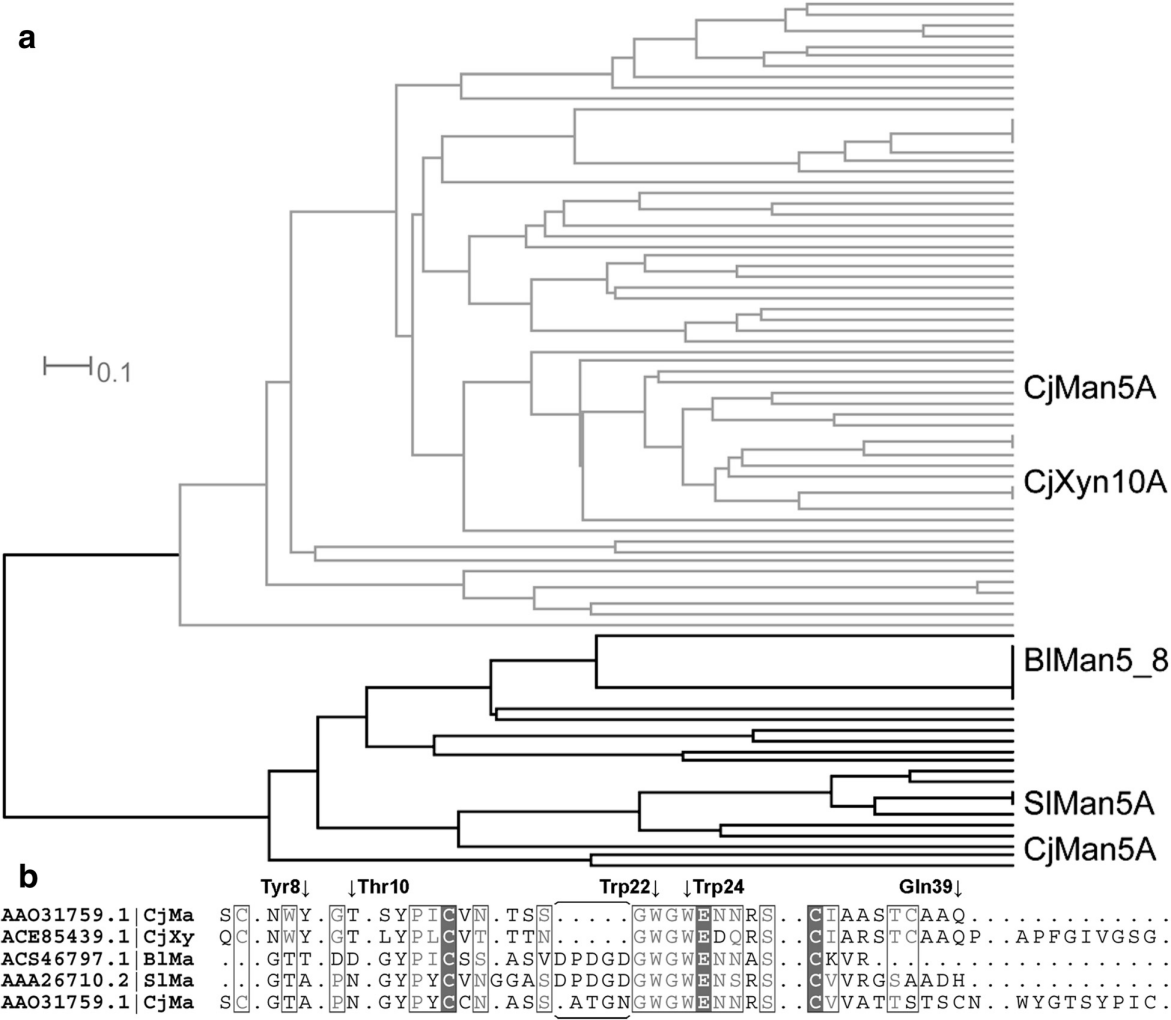
Phylogenetic tree and sequence alignment of CBM10 sequences. Phylogenetic tree constructed based on sequence alignment of available CBM10 sequences (**a**) and alignment of the CBM10 of *Bl*Man5_8 and putative CBM10 sequences of characterized enzymes as predicted by dbCAN (**b**). The sub-tree containing *Bl*Man5_8 is highlighted in black. The characterized enzymes *Bl*Man5_8, *Sl*Man5A [54], *Cj*Man5A [22] and *Cj*Xyn10A [55] are indicated. The dbCAN tool (http://csbl.bmb.uga.edu/dbCAN/index.php) was used to obtain putative CBM10 sequences and the sequence of the *Sl*Man5A CBM10 was added manually. Multiple sequence alignment of CBM10 from the five characterized enzymes is shown. The proposed binding residues Tyr^8^, Thr^10^, Trp^22^, Trp^24^ and Gln^39^ in the *Cj*Xyn10A CBM10 structure [52] are indicated by arrows. The unique insertion in the *Bl*Man5_8 clade preceding the binding residue Trp^22^ is indicated with a horizontal parenthesis

The CBM10 of *Bl*Man5_8 and close homologues thereof, which are exclusively joined to β-mannanase catalytic modules, populate a distinct clade in the CBM10 phylogenetic tree (Fig. 6A). This clade is distinguished by unique sequence features, including substitution of a tyrosine residue conserved in other CBM10s as well as an insertion of five residues (Fig. 6B).

## Discussion

The human gastrointestinal tract comprises one of the most densely populated ecological niches in nature, with bacterial counts exceeding 10^11^ cells · g^−1^ content [40]. Since most dietary glycans are indigestible to humans, their metabolism plays an instrumental role in shaping the composition of the gut microbiota [41]. To date, the GH26 mannanases from *Bacteroides fragilis* [42] and *Bifidobacterium adolescentis* [28] are the only described β-mannanases from the gut niche. In this study we report the biochemical properties of the first GH5 mannanase from the *Bifidobacterium* genus.

### *Bl*Man5_8 is an exceptionally potent enzyme optimized for efficient depolymerization of soluble mannan

The preferred substrates for *Bl*Man5_8 are soluble glucomannan and galactomannnan with low degree of galactosyl substitution (Table 1). *Bl*Man5_8 displays 19-fold higher *k*_cat_ on LBG as compared to M_5_ (Table 2), suggesting that substrate contacts beyond five mannosyl residues contribute to catalytic turnover, similarly to other mannanases [43]. The catalytic efficiency of *Bl*Man5_8 on LBG is the highest measured to date for a GH5 mannanase owing to both a very high *k*_cat_ and a low *K*_m_ (Additional file 11). Notably, the *K*_m_ value for *Bl*Man5_8 towards soluble mannan is one of the lowest amongst bacterial mannanases, which is likely to be an important advantage in the adaptation to the highly competitive gut ecological niche. The *K*_m_ value in part reflects substrate affinity, which appears higher at the aglycone-accommodating region of the active site cleft as evident from the productive binding frequency of M_5_ (Fig. 3). Substrate affinity at aglycone subsites has been shown to contribute to lowering *K*_m_ and increasing transglycosylation [30, 44]. This is in line with the high transglycosylation activity of *Bl*Man5_8, reflected by the disproportionate liberation of M_4_ relative to M_1_ ratio using M_5_ as a substrate (Fig. 2), which is attributed to secondary hydrolysis of transglycosylation products of larger degree of polymerization (Fig. 3). Interestingly, sequence comparison and homology modeling revealed that the putative aglycone binding regions of *Thermobifida fusca* GH5_8 mannanase (*Tf*Man5A) [34] and *Bl*Man5_8 are lined with conserved aromatic residues, including Trp^196^ and Trp^200^ (Additional file 10; Fig. 5). Indeed, the position and conservation of these residues correlate with the favored productive binding at the aglycone region (Fig. 4).

### The CBM10 of *Bl*Man5_8 binds soluble mannan with low affinity and represents a new clade within CBM10

The CBM10 of the modular *Cj*Xyn10A xylanase from *Cellvibrio japonicus*, which binds tightly to Avicel, is the only structurally characterized CBM10 member [21]. The Man5A β-mannanase from the same organism, which possesses a C-terminal tandem repeat of two CBM10 modules (*Cj*Man5A-CBM10-1-CBM10-2), was also shown to bind both Avicel and insoluble mannan (INM) [22]. Deletion of CBM10-2 of *Cj*Man5A, which is a close homolog to the counterpart from *Cj*Xyn10A, resulted in a severe reduction of affinity to Avicel and INM [22], which hinted that the CBM10-1 was not efficiently mediating binding to the above insoluble substrates. Similarly, the CBM10 of *Bl*Man5_8, which belongs to the same subgroup as the CBM10-1 of *Cj*Man5A, did not bind to Avicel or HEC. By contrast, this CBM10 conferred increased affinity to soluble mannan based on larger relative retardation in affinity electrophoresis of the full-length *Bl*Man5_8 compared to the truncated *Bl*Man5_8-ΔCBM10. Remarkably, the affinity of *Bl*Man5_8 for soluble galactomannan is 35 − 67-fold lower than that reported for GH26 mannanases *Ba*Man26A from *Bifidobacterium adolescentis* (*Ba*Man26A) [28] and *Cf*Man26A from *Cellulomonas fimi* [45] possessing typical mannan-specific CBM23 modules. Binding analysis using SPR confirmed the mannan specificity and low affinity of the CBM10 of *Bl*Man5_8, as deletion of this module considerably reduced the apparent stoichiometry of binding to M_5_ and M_6_ (Table 3). *Bl*Man5_8-ΔCBM10 moreover displayed about two-fold higher affinity to M_5_ and M_6_ than the full-length enzyme, suggesting that the CBM10 had lower affinity than the catalytic module. The CBM10 of *Bl*Man5_8 is a novel low-affinity CBM that binds manno-oligosaccharides with *K*_d_ values in the mM range, in contrast to canonical moderate-affinity mannan-binding CBMs displaying *K*_d_ values in the low μM range [46, 47]. Low-affinity starch-binding CBMs, conferring more dynamic binding, have been identified in plastidial α-amylases and starch-regulatory glucan water dikinases [48, 49].

The low affinity of the CBM10 is consistent with its very modest impact on the kinetic parameters of LBG hydrolysis (Table 2). The trend of increased *K*_m_ and *k*_cat_ upon CBM deletion is similar to other enzymes acting on soluble substrates, but the magnitude of these changes is much larger with typical CBMs than for the CBM10 of *Bl*Man5_8 [50, 51].

The CBM10 of *Bl*Man5_8 clusters exclusively with counterparts from GH5 and GH26 mannanases and segregates in the phylogenetic tree from sequences resembling the cellulose-specific CBM10 from *Cj*Xyn10A (Fig. 6; Additional file 12) also occurring in xylanases, cellulases and other carbohydrate-active enzymes as well as mannanases (Additional file 12). The aromatic binding residue Tyr^8^ in the cellulose-binding site of CBM10 of *Cj*Xyn10A [21] is substituted to smaller residues (Fig. 6, Additional file 9) in the *Bl*Man5_8 CBM10 subfamily, that possesses a unique insertion preceding the substrate binding residue Trp^22^ and lacks the two conserved cysteines Cys^5^ and Cys^38^ (Fig. 6) suggesting the loss of one disulphide bridge compared to the clade represented by the CBM10 of *Cj*Xyn10A [52] (Additional file 13). Altogether, the loss of the Tyr^8^ aromatic stacking platform combined with significant differences in the loop length close to the binding Trp^22^ provide a possible explanation for the low affinity of the CBM10 from *Bl*Man5_8 compared to *Cj*Xyn10A. Additionally, the loss of a disulfide bridge may elicit flexibility and/or structural changes that affect the biochemical properties of the *Bl*Man5_8 CBM10 and its close homologs. Assigning the effects of these differences requires further structural and mutational analyses of this clade of CBM10.

### Mannan utilization in the gut and the biological implication of *Bl*Man5_8 activity

The concentration and residence time of mannan in the gastrointestinal tract is expected to be highly dynamic and dependent on nutritional intake and gut microbiota composition. The presence of mannan utilization pathways in human gut-adapted taxa, including the major gut commensal genus *Bacteroides*, reflects an evolutionary adaptation to the dietary intake of this glycan. Recently, a novel pathway for the utilization of mannan involving a putative extracellular mannobiose-forming GH26 exo-mannanase from *Bacteroides fragilis* (*Bf*Man26A) was reported [42]. The mannobiose released by this enzyme is taken up by specialized ion symporters and subsequently degraded and metabolized intracellularly by an epimerase and a phosphorylase. This GH26 enzyme does not possess a CBM, and was speculated to act on mannan fragments produced by other β-mannanases from the same organism. The extracellular putatively cell-attached GH26 mannanase from *Bifidobacterium adolescentis* (*Ba*Man26A) hydrolyzes mannan to mainly mannotriose [28]. This modular enzyme, featuring a GH26 catalytic module joined to a C-terminal tandem repeat of CBM23 mannan-binding modules, binds tightly to LBG (*K*_d_ = 8.8 mg · L^−1^) [28]. The deletion of the tandem CBM23 repeat abolishes measurable binding and yields an enzyme with very high *K*_m_ (21.3 g · L^−1^), reminiscent of typical canonical moderate-affinity CBMs that decrease apparent *K*_m_ values of the enzymes they are attached to [50, 51]. This CBM-mediated decrease in *K*_m_, however, is often associated with a penalty of reduced *k*_cat_ [50, 51]. Such effects are very different in case of *Bl*Man5_8, where the CBM10 makes little contribution to the low *K*_m_. The justification for maintaining a CBM, albeit with lower affinity, could be a trade-off to increase the substrate-binding affinity of the enzyme (as observed from retardation electrophoresis data) and thus lower the *k*_off_ of the polymeric substrate, while minimizing the energy barrier pertaining to anchoring of the substrate tightly to the CBM. This may maintain proximity of the substrate to the enzyme, which has been shown to promote additional hydrolysis events following the initial enzyme-substrate encounter in cellulases [53] due to a higher *k*_cat_/*k*_off_ ratio. The optimization of substrate affinity through a low-affinity CBM is likely to offer a different adaptation solution to the fierce competition prevalent in the gut niche as opposed to the higher substrate affinity, but decrease in catalytic rates associated with canonical CBMs.

## Conclusions

In conclusion, *Bl*Man5_8, which is conserved in the *B. animalis* subsp. *lactis* displays a different modular organization, product profile and substrate preference than characterized β-mannanases from gut bacteria. *Bl*Man5_8 is the first characterized GH5 β-mannanase from the gut niche, which moreover possesses a novel low-affinity soluble-mannan-specific CBM10 not previously described in β-mannanases. This CBM may have evolved to increase the available substrate binding surface, while imparting less reduction in the catalytic rate typically observed for canonical moderate affinity CBMs. These unique substrate-binding properties highlight the diversity of mannan utilization strategies. Further studies are required to assess how these differences and the interplay of extracellular enzymes and transport systems contribute to establishing the hierarchy of mannan degradation in the gut niche.

## Additional files

Additional file 1:
**Composition of the polysaccharide substrates used in this study [** 56 **].** (PDF 50 kb) Additional file 2:
**Primers used for cloning of the full-length (** ***Bl*** **Man5_8) and generation of the truncated** (***Bl*** **Man5_8ΔCBM10) constructs.** (PDF 49 kb) Additional file 3:
**pH optimum (A) and stability (B) for** ***Bl*** **Man5_8 activity towards LBG. **pH stability is measured as relative activity after 4 days of storage in Britton-Robinson buffers of pH 2-10. Grey error bars represent deviation between duplicate samples. (TIF 1113 kb) Additional file 4:
**Temperature optimum (A) and Arrhenius plot (B) for** ***Bl*** **Man5_8-catalyzed hydrolysis of LBG.** Error bars represent deviation between duplicate samples. (TIF 1288 kb) Additional file 5:
**Baseline-corrected DSC thermogram of** ***Bl*** **Man5_8 unfolding.** Unfolding was measured by the normalized molar apparent heat capacity change (*C*_p_) as a function of temperature. (TIF 3356 kb) Additional file 6:
**Michaelis-Menten plots of (A) LBG and (B) M**_**5**_ **kinetics.** The initial normalized rate (*V*/E_0_) data for *Bl*Man5_8 and *Bl*Man5_8-ΔCBM10 are depicted as circles and triangles, respectively. The solid lines and the dashed lines depict the fits of the Michaelis-Menten equation to the initial normalized rate data for *Bl*Man5_8 and *Bl*Man5_8-ΔCBM10, respectively. (TIF 82 kb) Additional file 7:
**Affinity gel electrophoresis with LBG and HEC.** In all gels, BSA is in the left lane, *Bl*Man5_8 in the middle lane, and *Bl*Man5_8-ΔCBM10 in the right lane. (TIF 1882 kb) Additional file 8:
**Relative retardation of** ***Bl*** **Man5_8 in LBG-lv affinity electrophoresis.** Relative retardation is measured compared to BSA. The curve fitting was done with nonlinear regression using Prism 6 (GraphPad Software, La Jolla, CA, USA). (TIF 32 kb) Additional file 9:
**Sequence alignment of GH5 mannanases.** Multiple sequence alignment between characterized β-mannanases representative of different GH5 subfamilies, performed using MAFFT 7 and rendered with ESPript 3. The seven residues which are strictly conserved in all of GH5 [39] are marked with green boxes. Trp^196^, Trp^200^ and Tyr^225^, the three aromatic residues that are present in the putative aglycone subsites of *Bl*Man5_8, are also indicated. (TIF 29861 kb) Additional file 10:
**Multiple sequence alignment of GH5_8 sequences.** Included are sequences listed in CAZy as having UniProt entries. Positions corresponding to Trp^196^ and Trp^200^ in *Bl*Man5_8 (UniProt ID: C6A9J9) are indicated. The alignment was done with MAFFT 7 and rendered with ESPript 3. (TIF 17898 kb) Additional file 11:
**Comparison of kinetic parameters on LBG from characterized β-mannanases.** *k*_cat_ values have been re-calculated as s^-1^ rather than U · mg^-1^ where relevant [57–59]. (PDF 54 kb) Additional file 12:
**CBM10s of putative and characterized enzymes listed in dbCAN.** The list was manually curated by removing three CBM10 sequences with E-values above 0.02 and two which appeared fragmented, and adding the *Sl*Man5A (GenBank ID: AAA26710.2) CBM10 sequence. Characterized enzymes are indicated in bold. Enzymes with at least one CBM10 belonging to the same clade as the *Bl*Man5_8 CBM10 (GenBank ID: ACS46797.1) (Fig. 6) are highlighted with light grey background. (PDF 103 kb) Additional file 13:
**Multiple sequence alignment of characterized and predicted CBM10 sequences.** The list of CBM10 sequences was obtained from the annotation software website dbCAN (http://csbl.bmb.uga.edu/dbCAN/index.php) with the exception of the *Sl*Man5A CBM10 sequence which was added manually. The alignment was done with MAFFT 7 and rendered with ESPript 3. The positions corresponding to Cys^5^, Tyr^8^, Trp^22^, Trp^24^ and Cys^36^ in the *Cj*Xyn10A CBM10 structure [42] are indicated. The four cysteine residues form two disulphide bridges, while Tyr^8^, Trp^22^ and Trp^24^ where shown to mediate binding to insoluble cellulose in the *Cj*Xyn10A CBM10. (TIF 20792 kb)

## Abbreviations

Avicel: Microcrystalline cellulose
CAZy: Carbohydrate-Active enZYmes database
CBM: Carbohydrate-binding module
GG: Guar gum
GH: Glycoside hydrolase
HEC: Hydroxyethyl cellulose
HPAEC-PAD: High-performance anion exchange chromatography with pulsed amperometric detection
INM: Ivory nut mannan
KGM: Konjac glucomannan
LBG: Locust bean (carob) galactomannan
LBG-lv: Low-viscosity locust bean galactomannan
M_1_: Mannose
M_2_: Mannobiose
M_3_: Mannotriose
M_4_: Mannotetraose
M_5_: Mannopentaose
M_6_: Mannohexaose
M_7_: Mannoheptaose
M_8_: Mannooctaose
RU: Response units
SPR: Surface plasmon resonance
