# Supplementary material for: The GH5 1,4-β-mannanase from Bifidobacterium animalis subsp. lactis Bl-04 possesses a low-affinity mannan-binding module and highlights the diversity of mannanolytic enzymes
Source: BMC Biochem. 2015 Nov 11;16:26. doi: 10.1186/s12858-015-0055-4 (PMC4642672; doi:10.1186/s12858-015-0055-4)
Supplement: Additional file 1: — Composition of the polysaccharide substrates used in this study [ 56 ]. (PDF 50 kb) [file 12858_2015_55_MOESM1_ESM.pdf]

| **Substrate** | **Backbone** | **Side groups** | **Gal:Man ratio**[a] |
|---|---|---|---|
| LBG | β-1,4-Mannosyl | α-1,6-Galactosyl | 1:4 |
| LBG-lv | β-1,4-Mannosyl | α-1,6-Galactosyl | 1:4 |
| GG | β-1,4-Mannosyl | α-1,6-Galactosyl | 1:2 |
| INM[c] | β-1,4-Mannosyl | – | – |
| KGM | β-1,4-Mannosyl, β-1,4-glucosyl[b] | Acetyl | – |
| Avicel[c] | β-1,4-Glucosyl | – | – |

[a] Galactose:mannose ratio, defined as the average number of side group units per backbone unit.
[b] The mannose:glucose ratio of KGM is approximately 1.6:1 [56].
[c] INM and Avicel are water-insoluble crystalline or microcrystalline substrates.
