# Supplementary material for: The GH5 1,4-β-mannanase from Bifidobacterium animalis subsp. lactis Bl-04 possesses a low-affinity mannan-binding module and highlights the diversity of mannanolytic enzymes
Source: BMC Biochem. 2015 Nov 11;16:26. doi: 10.1186/s12858-015-0055-4 (PMC4642672; doi:10.1186/s12858-015-0055-4)
Supplement: Additional file 2: — Primers used for cloning of the full-length ( Bl Man5_8) and generation of the truncated (Bl Man5_8ΔCBM10) constructs. (PDF 49 kb) [file 12858_2015_55_MOESM2_ESM.pdf]

Construct	Primer	Sequence
*Bl*Man5_8	sense[a]	CTAGCTAGCGCGGACAATTCGCTCCACGTC
	antisense[a]	CGGGATCCTCAGCGAACCTTGCAGCTCGC
*Bl*Man5_8-ΔCBM10	sense[b]	CTCCAATTCCGGTGGCT***A***GGGTAACACTGG
	antisense[b]	CCAGTGTTACCC***T***AGCCACCGGAATTGGAG
[a] The primers are from the 5' ends and the restriction sites are underlined.<br>[b] The mutated basepair at position 1013 is underlined and highlighted in italics.		
