# Supplementary figures and images for: The GH5 1,4-β-mannanase from Bifidobacterium animalis subsp. lactis Bl-04 possesses a low-affinity mannan-binding module and highlights the diversity of mannanolytic enzymes
Source: BMC Biochem. 2015 Nov 11;16:26. doi: 10.1186/s12858-015-0055-4 (PMC4642672; doi:10.1186/s12858-015-0055-4)

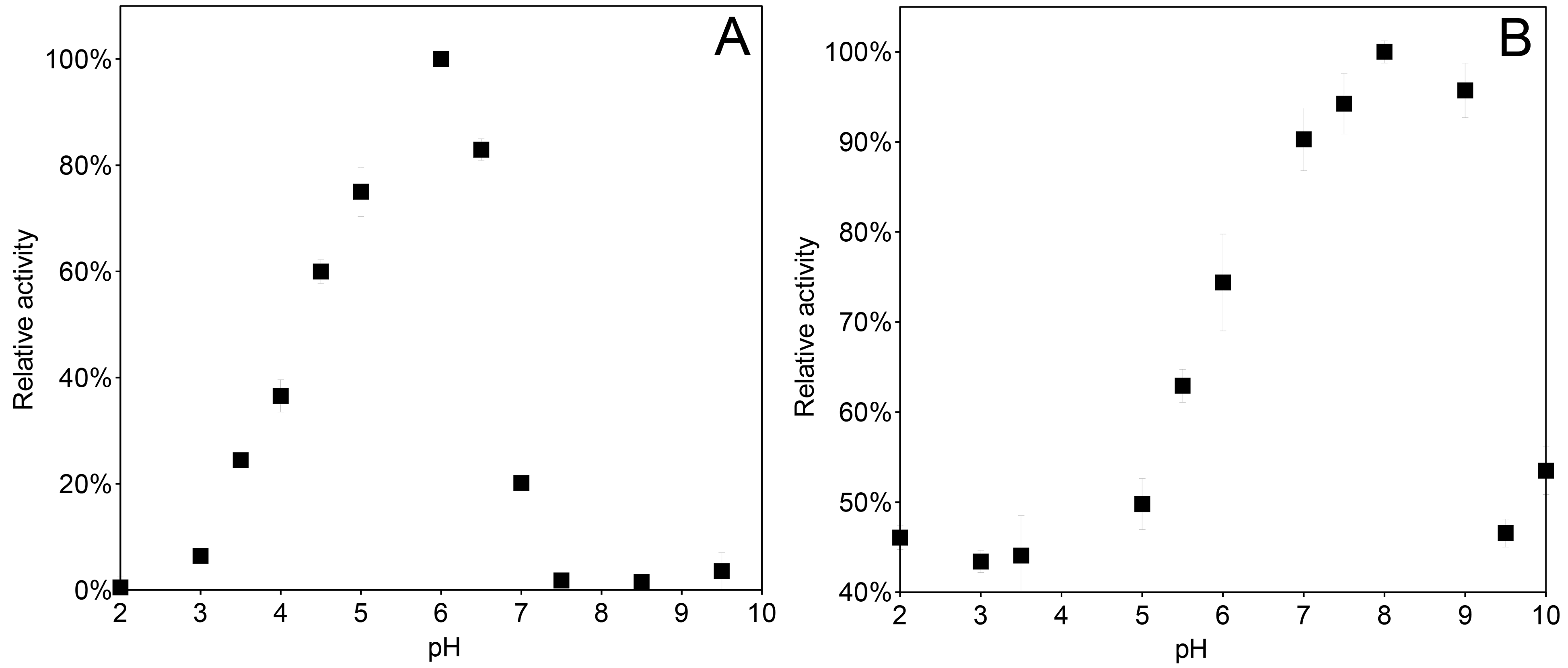

Supplement: Additional file 3: — pH optimum (A) and stability (B) for Bl Man5_8 activity towards LBG. pH stability is measured as relative activity after 4 days of storage in Britton-Robinson buffers of pH 2-10. Grey error bars represent deviation between duplicate samples. (TIF 1113 kb) [file 12858_2015_55_MOESM3_ESM.tif]

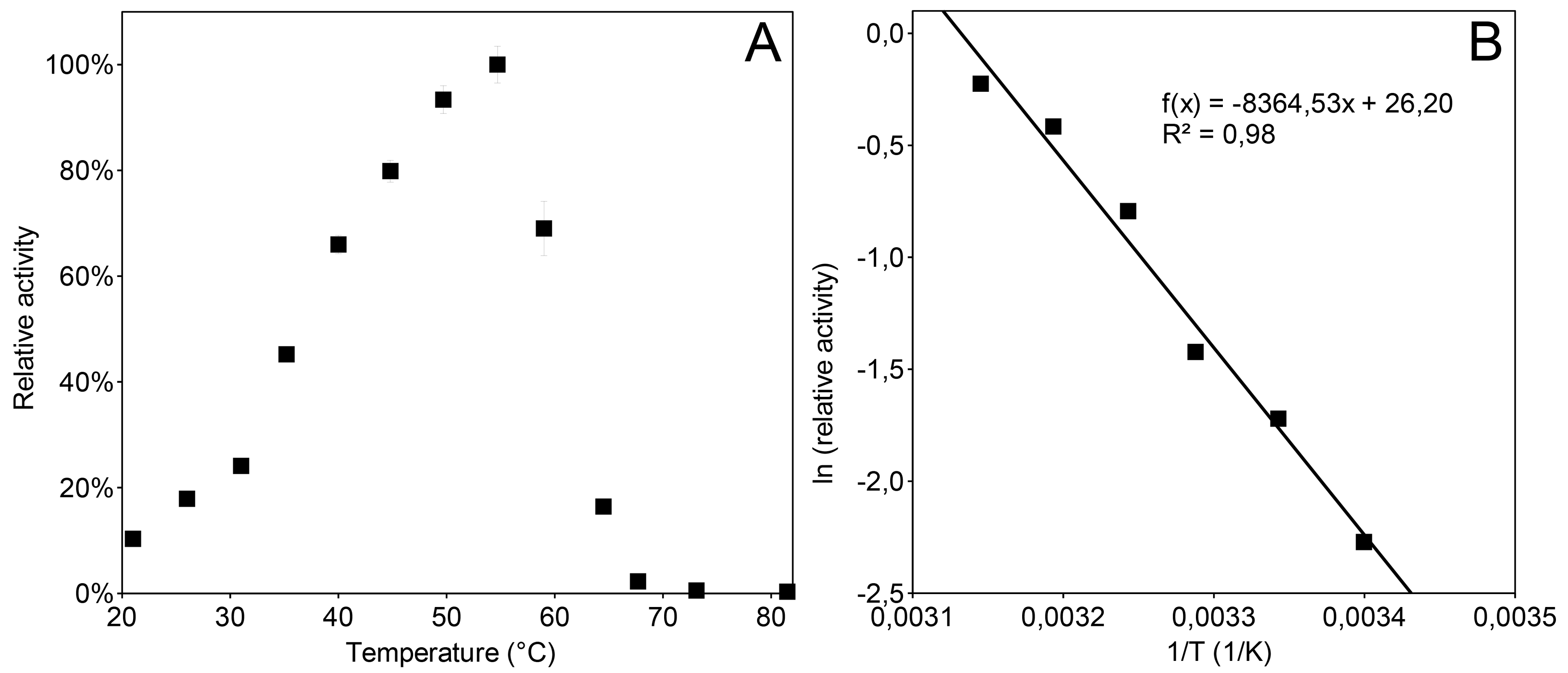

Supplement: Additional file 4: — Temperature optimum (A) and Arrhenius plot (B) for Bl Man5_8-catalyzed hydrolysis of LBG. Error bars represent deviation between duplicate samples. (TIF 1288 kb) [file 12858_2015_55_MOESM4_ESM.tif]

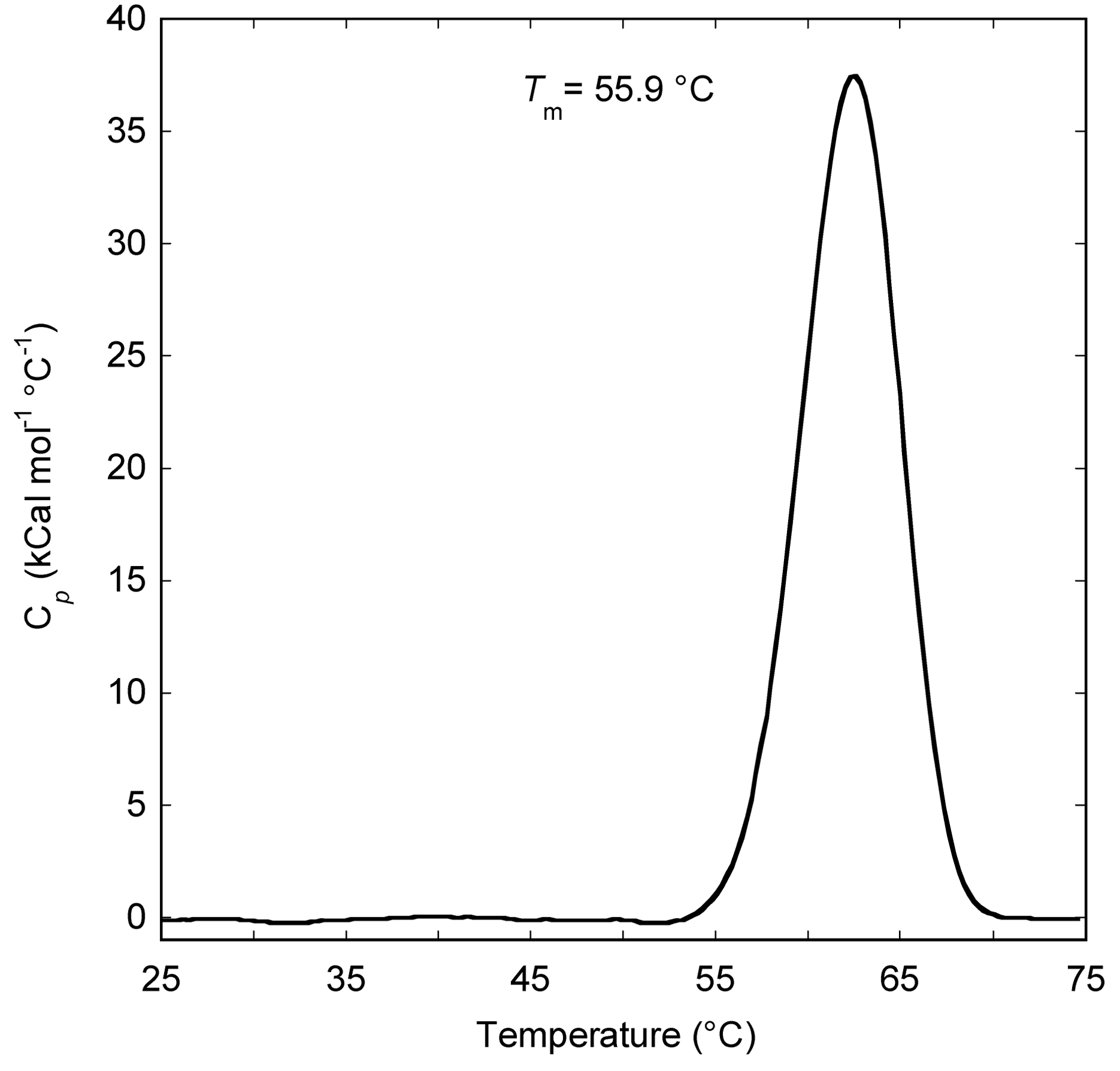

Supplement: Additional file 5: — Baseline-corrected DSC thermogram of Bl Man5_8 unfolding. Unfolding was measured by the normalized molar apparent heat capacity change (C p) as a function of temperature. (TIF 3356 kb) [file 12858_2015_55_MOESM5_ESM.tif]

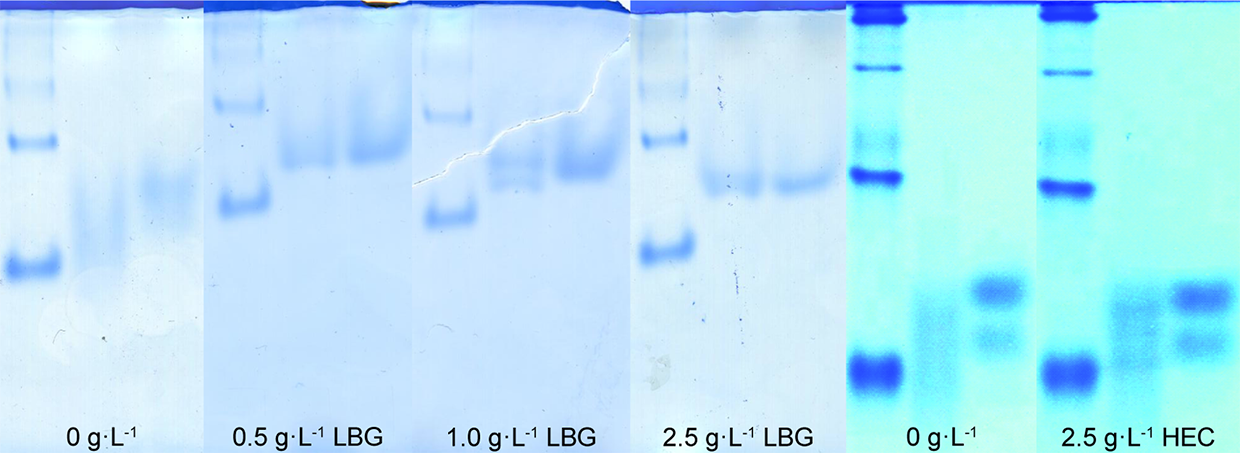

Supplement: Additional file 7: — Affinity gel electrophoresis with LBG and HEC. In all gels, BSA is in the left lane, BlMan5_8 in the middle lane, and BlMan5_8-ΔCBM10 in the right lane. (TIF 1882 kb) [file 12858_2015_55_MOESM7_ESM.tif]

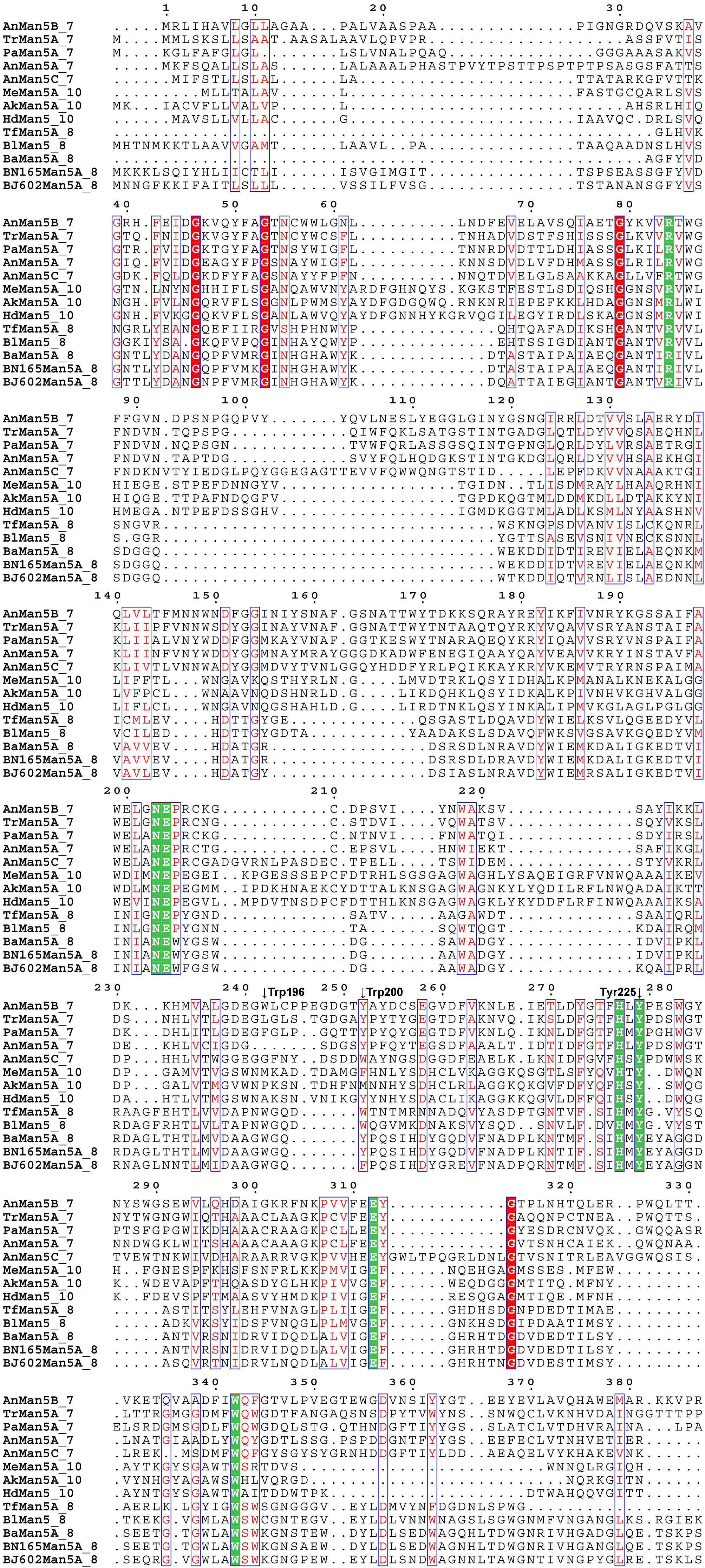

Supplement: Additional file 9: — Sequence alignment of GH5 mannanases. Multiple sequence alignment between characterized β-mannanases representative of different GH5 subfamilies, performed using MAFFT 7 and rendered with ESPript 3. The seven residues which are strictly conserved in all of GH5 [39] are marked with green boxes. Trp196, Trp200 and Tyr225, the three aromatic residues that are present in the putative aglycone subsites of BlMan5_8, are also indicated. (TIF 29861 kb) [file 12858_2015_55_MOESM9_ESM.tif]

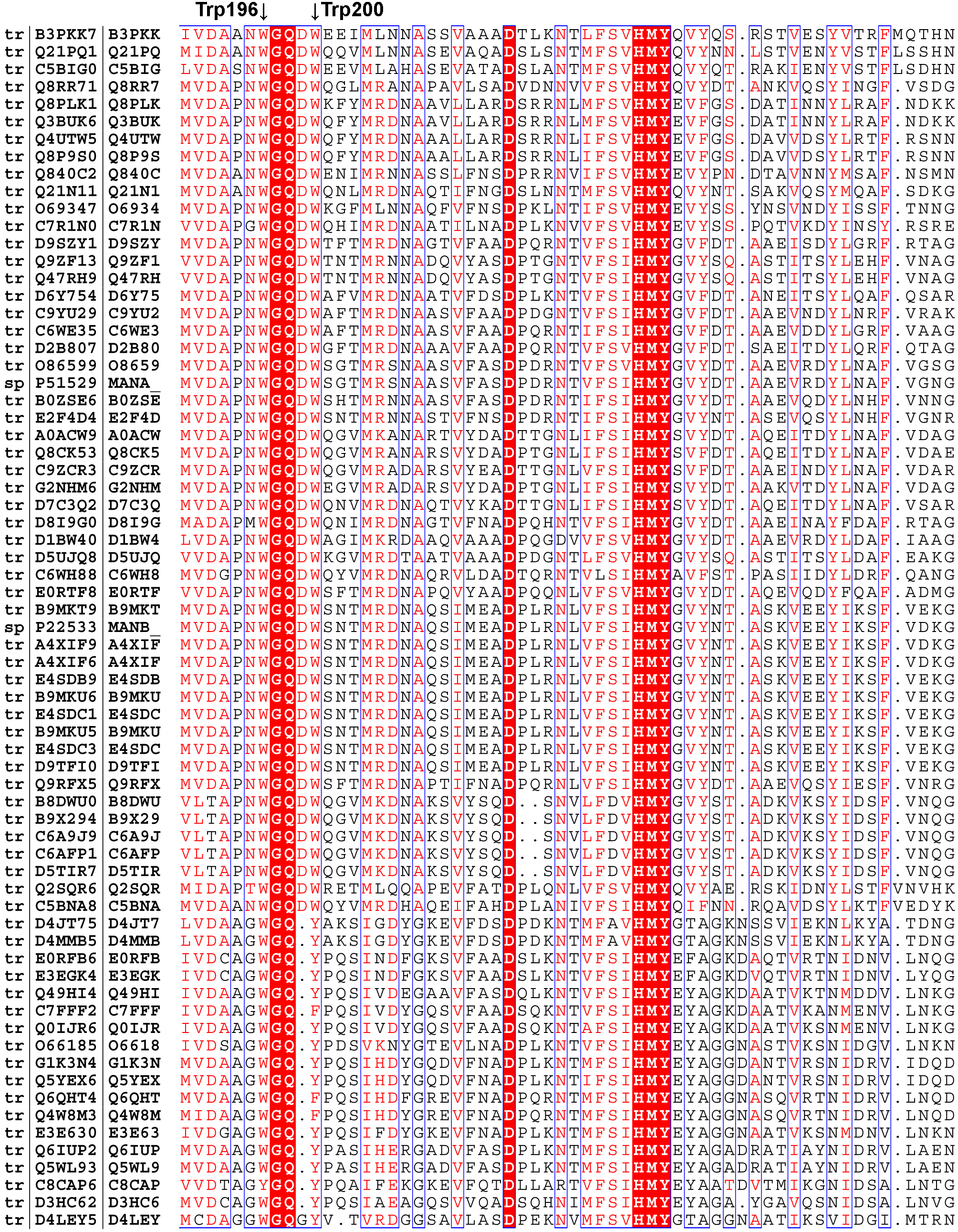

Supplement: Additional file 10: — Multiple sequence alignment of GH5_8 sequences. Included are sequences listed in CAZy as having UniProt entries. Positions corresponding to Trp196 and Trp200 in BlMan5_8 (UniProt ID: C6A9J9) are indicated. The alignment was done with MAFFT 7 and rendered with ESPript 3. (TIF 17898 kb) [file 12858_2015_55_MOESM10_ESM.tif]

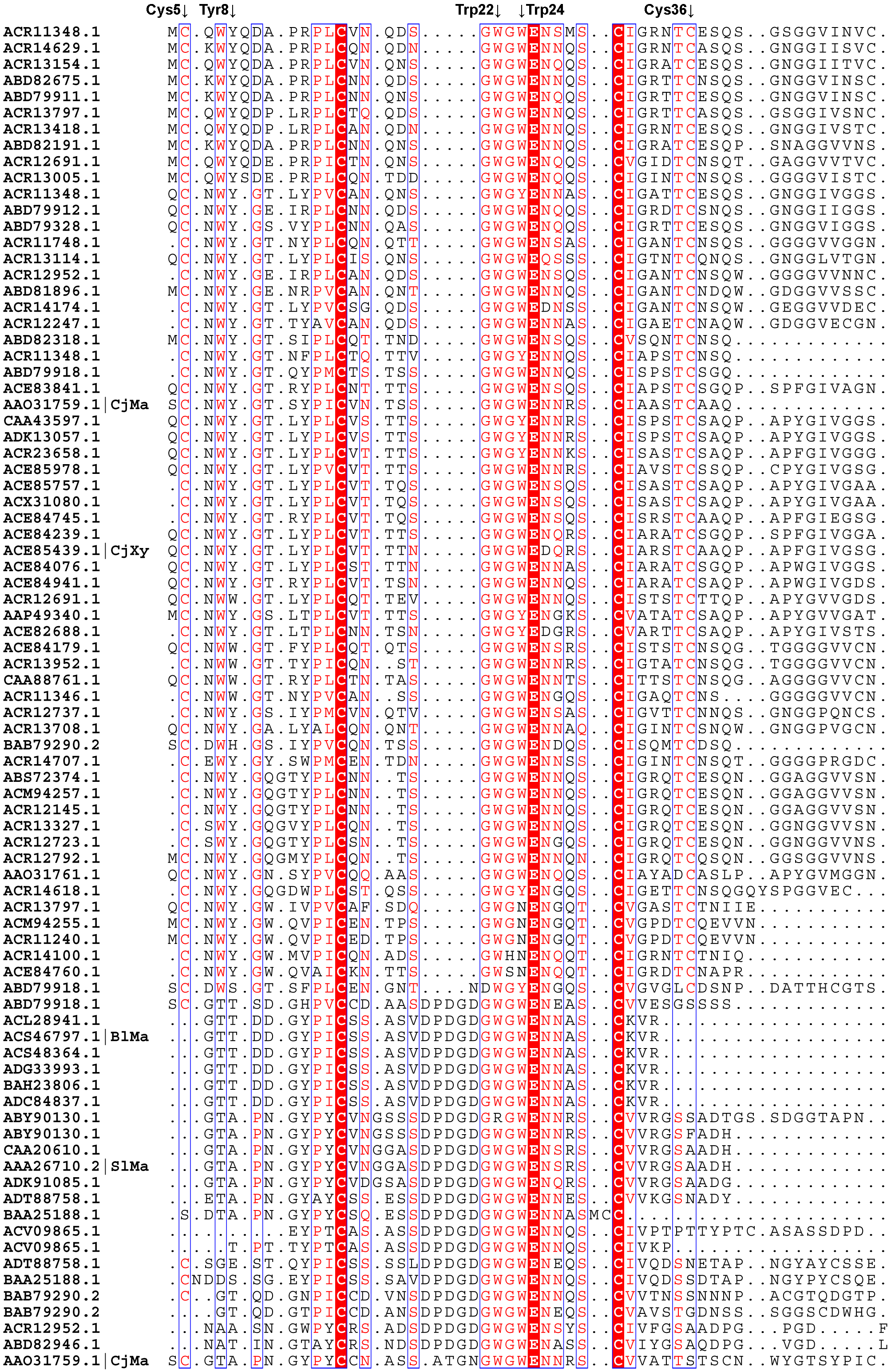

Supplement: Additional file 13: — Multiple sequence alignment of characterized and predicted CBM10 sequences. The list of CBM10 sequences was obtained from the annotation software website dbCAN (http://csbl.bmb.uga.edu/dbCAN/index.php) with the exception of the SlMan5A CBM10 sequence which was added manually. The alignment was done with MAFFT 7 and rendered with ESPript 3. The positions corresponding to Cys5, Tyr8, Trp22, Trp24 and Cys36 in the CjXyn10A CBM10 structure [42] are indicated. The four cysteine residues form two disulphide bridges, while Tyr8, Trp22 and Trp24 where shown to mediate binding to insoluble cellulose in the CjXyn10A CBM10. (TIF 20792 kb) [file 12858_2015_55_MOESM13_ESM.tif]
