# Supplementary material for: The GH5 1,4-β-mannanase from Bifidobacterium animalis subsp. lactis Bl-04 possesses a low-affinity mannan-binding module and highlights the diversity of mannanolytic enzymes
Source: BMC Biochem. 2015 Nov 11;16:26. doi: 10.1186/s12858-015-0055-4 (PMC4642672; doi:10.1186/s12858-015-0055-4)
Supplement: Additional file 11: — Comparison of kinetic parameters on LBG from characterized β-mannanases. k cat values have been re-calculated as s-1 rather than U · mg-1 where relevant [57–59]. (PDF 54 kb) [file 12858_2015_55_MOESM11_ESM.pdf]

| **Enzyme** | $k_{cat}$ **(s⁻¹)** | $K_m$ **(g·L⁻¹)** | $k_{cat}/K_m$ **(L·g⁻¹·s⁻¹)** | **Reference** |
|---|---|---|---|---|
| *Bl*Man5_8 | 1828 | 1.58 | 1157 | Current study |
| *Bl*Man5_8-ΔCBM10 | 2005 | 1.75 | 1146 | Current study |
| *Tr*Man5A | 240 | 0.6 | 400 | [30] |
| *Tr*Man5A-R171K | 260 | 1.3 | 200 | [30] |
| *Sl*Man5A | 197 | 3.5 | 56 | [54] |
| *Sl*Man5A-ΔCBM | 139 | 4.3 | 32 | [54] |
| *An*Man5A | 330 | 2.0 | 165 | [57] |
| *As*Man5A | 276 | 0.93 | 297 | [58] |
| *Cj*Man5A | 2549 | 13.9 | 216 | [22] |
| *Cj*Man5B | 125 | 3.0 | 38 | [22] |
| *Cj*Man5C | 4.1 | 8.4 | 0.53 | [22] |
| *Rm*Man26A | 2702 | 1.9 | 1422 | [59] |
| *Ba*Man26A-53K | 444 | 21.3 | 21 | [28] |
| *Cj*Man26B | 259 | 3.3 | 92 | [22] |
