# Supplementary material for: The GH5 1,4-β-mannanase from Bifidobacterium animalis subsp. lactis Bl-04 possesses a low-affinity mannan-binding module and highlights the diversity of mannanolytic enzymes
Source: BMC Biochem. 2015 Nov 11;16:26. doi: 10.1186/s12858-015-0055-4 (PMC4642672; doi:10.1186/s12858-015-0055-4)
Supplement: Additional file 12: — CBM10s of putative and characterized enzymes listed in dbCAN. The list was manually curated by removing three CBM10 sequences with E-values above 0.02 and two which appeared fragmented, and adding the SlMan5A (GenBank ID: AAA26710.2) CBM10 sequence. Characterized enzymes are indicated in bold. Enzymes with at least one CBM10 belonging to the same clade as the BlMan5_8 CBM10 (GenBank ID: ACS46797.1) (Fig. 6) are highlighted with light grey background. (PDF 103 kb) [file 12858_2015_55_MOESM12_ESM.pdf]

GenBank ID	Enzyme	Family	Organism	CBM10s
**AAA26710.2**	**β-1,4-mannanase Man5A**	**GH5**	***Strepromyces lividans***	**1**
**AAO31759.1**	**β-1,4-mannanase Man5A**	**GH5**	***Cellvibrio japonicus***	**2**
**AAO31761.1**	**β-1,4-mannanase Man5C**	**GH5**	***Cellvibrio japonicus***	**1**
AAP49340.1	GnuB	GH45	Uncultured bacterium	1
ABD79328.1	Endoglucanase	GH5	*Saccharophagus degradans*	1
ABD79911.1	Endoglucanase	GH9	*Saccharophagus degradans*	1
ABD79912.1	Putative cellulose-binding protein	Unknown	*Saccharophagus degradans*	1
ABD79918.1	Putative β-mannanase	GH5	*Saccharophagus degradans*	3
ABD81896.1	Endoglucanase	GH5	*Saccharophagus degradans*	1
ABD82191.1	β-xylanase	GH10	*Saccharophagus degradans*	1
ABD82318.1	Putative bifunctional xylanase / acetylxylan esterase	GH11	*Saccharophagus degradans*	1
ABD82675.1	Endoglucanase	GH5	*Saccharophagus degradans*	1
ABD82946.1	Putative β-mannanase	GH26	*Saccharophagus degradans*	1
**ABS72374.1**	**Bifunctional β-1,4-endoglucanase / cellobiohydrolase**	**GH5 / 6**	***Teredinibacter turnerae***	**1**
ABY90130.1	β-1,4-mannanase	GH5	*Streptomyces* sp. s6-204	2
**ACE82688.1**	**Endoglucanase B**	**GH45**	***Cellvibrio japonicus***	**1**
ACE83841.1	Endoglucanase	GH5	*Cellvibrio japonicus*	1
ACE84076.1	Endoglucanase	GH5	*Cellvibrio japonicus*	1
ACE84179.1	β-1,4-xylanase Xyn11A	GH11	*Cellvibrio japonicus*	1
ACE84239.1	Cellulose or protein binding domain	Unknown	*Cellvibrio japonicus*	1
ACE84745.1	β-1,4-glucanase / xyloglucanase, putative	GH74	*Cellvibrio japonicus*	1
ACE84760.1	Chitin binding protein, putative	Unknown	*Cellvibrio japonicus*	1
ACE84941.1	Endoglucanase	GH5	*Cellvibrio japonicus*	1
**ACE85439.1**	**β-1,4-xylanase A**	**GH10**	***Cellvibrio japonicus***	**1**
ACE85757.1	Endoglucanase A	GH9	*Cellvibrio japonicus*	1
ACE85978.1	Glucanase	GH6	*Cellvibrio japonicus*	1
ACL28941.1	β-1,4-mannanase	GH5	*Bifidobacterium animalis* subsp. *lactis* AD011	1
ACM94255.1	Unidentified	Unknown	*Bankia gouldi*	1
ACM94257.1	Unidentified	Unknown	*Teredinibacter*	1
ACR11240.1	Endoglucanase	GH9	*Teredinibacter turnerae*	1
ACR11346.1	Xylanase / acetylxylan esterase	GH11	*Teredinibacter turnerae*	1
ACR11348.1	GH5 domain protein	GH5	*Teredinibacter turnerae*	3
ACR11748.1	β-xylanase	GH10	*Teredinibacter turnerae*	1
ACR12145.1	GH5 and GH6 domain protein	GH5 / 6	*Teredinibacter turnerae*	1
ACR12247.1	Endoglucanase	GH5	*Teredinibacter turnerae*	1
ACR12691.1	Arabinogalactan endo-β-1,4-galactanase	GH53	*Teredinibacter turnerae*	2
ACR12723.1	Glucanase	GH6	*Teredinibacter turnerae*	1
ACR12737.1	GH16 domain protein	GH16	*Teredinibacter turnerae*	1
ACR12792.1	GH11 and GH5 domain protein	GH11 / 5	*Teredinibacter turnerae*	1
ACR12952.1	GH26 domain protein[a]	GH26	*Teredinibacter turnerae*	2
ACR13005.1	Cellulase	GH45	*Teredinibacter turnerae*	1
ACR13114.1	GH16 domain protein	GH16	*Teredinibacter turnerae*	1
ACR13154.1	Carbohydrate esterase, family3	CE3	*Teredinibacter turnerae*	1
ACR13327.1	GH5 domain protein	GH5	*Teredinibacter turnerae*	1
ACR13418.1	β-xylanase	GH10	*Teredinibacter turnerae*	1
ACR13708.1	Glucose / sorbosone dehydrogenase domain protein	Unknown	*Teredinibacter turnerae*	1
ACR13797.1	Endoglucanase	GH5	*Teredinibacter turnerae*	2

ACR13952.1	Xylanase / acetylxylan esterase	GH11	*Teredinibacter turnerae*	1
ACR14100.1	CBM33 and 10 domain protein	Unknown	*Teredinibacter turnerae*	1
ACR14174.1	GH74 domain protein	GH74	*Teredinibacter turnerae*	1
ACR14618.1	O-Glycosyl hydrolase family 30	GH30	*Teredinibacter turnerae*	1
ACR14629.1	Endoglucanase	GH9	*Teredinibacter turnerae*	1
ACR14707.1	Gluconolactolase domain protein	Unknown	*Teredinibacter turnerae*	1
ACR23658.1	Endoglucanase	GH9	Bacterium enrichment culture clone CelA20	1
**ACS46797.1**	**Secreted β-mannosidase (mannanase)**	**GH5**	***Bifidobacterium animalis* subsp. *lactis* Bl-04**	**1**
ACS48364.1	Secreted β-mannosidase (mannanase)	GH5	*Bifidobacterium animalis* subsp. *lactis* DSM 10140	1
ACV09865.1	GH5 domain protein[a]	GH5	*Jonesia denitrificans*	2
ACX31080.1	Endoglucanase	GH9	*Pseudomonas cellulosa*	1
ADC84837.1	β-1,4-mannanase	GH5	*Bifidobacterium animalis* subsp. *lactis* BB-12	1
ADG33993.1	Secreted β-mannosidase (mannanase)	GH5	*Bifidobacterium animalis* subsp. *lactis* V9	1
ADK13057.1	Endoglucanase	GH5	*Pseudomonas fluorescens*	1
**ADK91085.1**	**β-1,4-mannanase**	**GH5**	***Streptomyces* sp. S27**	**1**
ADT88758.1	β-1,4-mannanase	GH5	*Vibrio furnissii*	2
BAA25188.1	β-1,4-mannanase	GH5	*Vibrio* sp. MA138	2
BAB79290.2	β-1,4-mannanase	GH5	*Pseudomonas* sp. ND137	3
BAH23806.1	β-1,4-mannanase	GH5	*Bifidobacterium animalis* subsp. *lactis*	1
CAA20610.1	Secreted β-mannosidase (mannanase)	GH5	*Streptomyces coelicolor*	1
**CAA43597.1**	**Endoglucanase C**	**GH5**	***Cellvibrio japonicus***	**1**
CAA88761.1	β-1,4-xylanase	GH11	*Cellvibrio mixtus*	1

[a] Predicted β-mannanases according to BLAST analysis.
